# Application of CZE Method in Routine Analysis for Determination of B-Complex Vitamins in Pharmaceutical and Veterinary Preparations

**DOI:** 10.1155/2012/592650

**Published:** 2012-03-22

**Authors:** Marina Franco, Renata Jasionowska, Elisa Salvatore

**Affiliations:** Dipartimento di Chimica, Sapienza Università, 00185 Roma, Italy

## Abstract

A competitive CZE method for quality control analysis of multivitamin preparations and veterinary products containing B-group vitamins was developed. Vitamins of interest are thiamine hydrochloride (B_1_), thiamine monophosphate chloride (B_1a_), riboflavine (B_2_), riboflavine-5′monophosphate (B_2a_), nicotinamide (B_3_), d-pantothenic acid calcium salt (B_5_), pyridoxine hydrochloride (B_6_), folic acid (B_9_), and 4-aminobenzoic acid (B_10_). These analytes were separated optimizing the experimental conditions in 20 mM tetraborate buffer pH = 9.2 as a BGE (background electrolyte), on a Beckman P/ACE System MDQ instrument, using uncoated fused silica capillary. The effective capillary length was of 49.5 cm, I.D. = 50 *μ*m, the applied voltage 20 kV and the temperature 25°C. Detection was performed by a diode array detector at 214 nm for all vitamins except B_5_ (190 nm) and B_2a_ (260 nm). Separation time was about 9 min. After experimental conditions optimization, the proposed method was validated. Precision of migration time and corrected peak area, linearity range, LOD and LOQ, accuracy (recovery), robustness, and ruggedness were evaluated for each analyte demonstrating the good reliability of the method. Analyses of the pharmaceutical real samples were performed and confirmed the versatility of this method.

## 1. Introduction

Quality control (QC) plays an essential role in the pharmaceutical industry. In fact many analytical methods, employing different techniques, are being developed for evaluation of pharmaceutical preparations. Vitamins of B group were well separated by RP-HPLC. A method for the simultaneous determination of taurine and 10 water-soluble vitamins including vitamin B_1_ (thiamine), B_2_ (riboflavine), B_5_ (pantothenic acid), B_6_ (pyridoxine and pyridoxal), B_8_ (biotin), B_9_ (folic acid), C (ascorbic acid), and B_3_ (nicotinamide and nicotinic acid) in multivitamin tablets was developed and validated. Detection of components was by ESI-MS [1]. Some water-soluble vitamins of our interest (ascorbic acid, thiamine hydrochloride, riboflavine-5′-phosphate sodium, pyridoxine hydrochloride, nicotinamide, and (+)-panthenol) and two preservatives (methylparaben and sodium benzoate) in multivitamin syrup were well-separated on Zorbax SB-Aq (C18) column [2]. A HPLC-UV method was established for the simultaneous determination of eight vitamins, including B_1_, B_2_, B_3_, B_6_, B_9_, cyanocobalamin, ascorbic acid in baby milk powder [3]. A validated HPLC-UV method for the determination of seven B-complex vitamins (B_1_, B_2_, B_3_, B_6_, B_9_, and cyanocobalamin) in pharmaceuticals and biological fluids after solid-phase extraction was performed [4]. Simultaneous determination of water- and fat-soluble vitamins in pharmaceutical preparations by HPLC was performed in a single run using combined isocratic and linear gradient elution with a mobile phase consisting of trifluoroacetic acid and methanol. The method was applied for real sample. The results were in good agreement with the declared values. Vitamins analyzed were B_1_, B_2_, B_5_, B_6_, B_9_, B_3_, and B_12_ (cyanocobalamin) [5].

The RP ion-pair HPLC method was applied for determination of some vitamins of our interest in multivitamin with minerals from different authors. For nicotinamide, pyridoxine hydrochloride, thiamine mononitrate and riboflavine a HPLC method was validated using methanol 0.5% acetic acid as the mobile phase [6].

A sensitive RP-HPLC method was developed and validated for the simultaneous determination of B_1_, B_3_, B_6_, and B_9_ in Pentovit coated tablets. The procedures were carried out on a Supelcosil ABZ column with methanol, heptanesulfonic acid sodium salt, and triethylamine as the mobile phase [7]. For the determination of thiamine and riboflavine in Duoweiyuansu Tablet an HPLC-UV method was established. The mobile phase consisted of ion-pairing reagent (containing 1-hexanesulfonate, glacial acetic acid, and triethylamine)/methanol (80 : 20). The method can be used for the quality control of thiamine and riboflavine in Duoweiyuansu Tablet [8]. There are many papers about vitamins of B group analysed by capillary electrophoresis (CE) and its modifications. Some authors studied determination of vitamins in food based on supercritical fluid extraction prior to micellar electrokinetic capillary chromatographic (MEKC) analyses of individual components. The method was optimized using sodium cholate as the micellar phase for the separation of 11 B-vitamins, ascorbic acid, and 4 impurities in about 25 min [9]. Other authors investigated in capillary zone electrophoresis (CZE) analysis the effect of lower organic alcohols as cosurfactants (methanol, ethanol, n-propanol, isopropanol, propanediol, n-butanol and isoamyl alc.) and n-hexane as an organic modifier in phosphate buffer with varying SDS concentration using a set of vitamins and p-hydroxybenzoic acid as the test mixture. Optimum separations were achieved particularly at high concentrations of the surfactant [10]. The CZE analyses of three vitamins (B_1_, B_2_, and B_6_ in tablets achieved using phosphate-borate buffer pH 9.0 short time of analysis (5 min) [11]. One year later the same authors studied another specific, precise, sensitive, and accurate method for separation of the same vitamins obtaining high separation efficiency and shorter analysis time (3 min) [12]. Four B vitamins, as B_1_, B_2_, pyridoxal, pyridoxine, and pyridoxamine (B_6_) in a pharmaceutical product were determined simultaneously using CZE. An HCl soln. was used for the extraction of the vitamins from a multivitamin-multimineral tablet. BGE employed was sodium phosphate buffer (pH 9.0) [13].

Mixture of four water-soluble B-group vitamins were analysed both by CZE and MECK. The quantitative analyses of different pharmaceutical formulations were compared with the LC method of the US Pharmacopeia obtaining a good correlation [14].

Rapid methods were developed for simultaneously separation of five vitamins: B_1_, B_2_, B_6_, nicotinamide, nicotinic, and ascorbic acid. They were tested on 15 real samples obtaining good resolution by (CZE) and (MECC). CZE was performed with 0.02 M borate buffer, while MECC in 0.02 M borate/phosphate buffer with 4% acetonitrile containing. 0.1 M sodium dodecyl sulfate [15].

Some authors studied a method for the analysis of six water-soluble vitamins (thiamine, nicotinamide, riboflavin, pyridoxine, pantothenic, and ascorbic acid) in a pharmaceutical formulation, by CZE. A good compromise between resolution, analysis time, and analyte stability was obtained by use of a 50 mM borax buffer of pH 8.5. This CZE method was very useful for the separation of more complex samples, but cyanocobalamin could not be separated from nicotinamide in this CZE system. In fact the 2 compounds were in uncharged form the pH used. Instead, they were resolved by MECC using SDS in BGE. Good results with respect to linearity, precision, and accuracy were obtained in the concentration range studied for the 6 vitamins [16].

There are some studies about B-complex by spectrophotometric methods.

Spectrophotometric determination of ternary mixtures of thiamine, riboflavin, and pyridoxal in pharmaceutical and human plasma by least-squares support vector machines were performed. The partial least squares (PLS) modelling and least-squares support vector machines were used for the multivariate calibration of the spectrophotometric data [17]. The same procedure was applied successfully for determination of B_1_, B_2_, B_3_, and B_6_ in pharmaceuticals by other authors [18].

Simultaneous determination of fourteen water-soluble vitamins (13 of B-group and vitamin C) in selected food matrices by LC/MS/MS technique is described in a recent paper [19]. Analytes were separated in less than 10 min with recoveries between 30% and 70%.

The QC process in pharmaceutical industry needs methods that are able to determine, in a single run, the majority of components. In many cases, to determine all components of a multivitamin pharmaceutical preparation, it is necessary to perform different analyses applying different techniques.

Capillary electrophoresis (CE) is a powerful analytical technique that is widely used in research, and in quality control of pharmaceuticals. As demonstrated exploring the literature data, the MEKC was the only method [9] able to separate up to 11 B-group vitamins. Other CE methods offer the possibility to separate only a few (max 5) molecules of this class. There are no publications about simultaneous determination of nine B-group vitamins by CZE, the simplest in CE system, which offers several advantages over HPLC such as rapid analysis, lower solvent consumption, then lower costs and minor environmental impact, and higher efficiency.

For this purpose, the aim of this work is to develop a validated CZE method for assay of a multicomponent pharmaceutical and veterinary formulations in routine analysis.

## 2. Experimental

### 2.1. Apparatus

The analyses were carried out on a P/ACE system from Beckman Instrument Fullerton, CA (USA), with a UV-DAD detector. For ruggedness evaluation, electrophoretic separations were carried out on the SpectraPhoresis 1000 instrument from Thermo-Quest Corporation, CA (USA).

The uncoated fused silica capillary ID = 50 *μ*m, 59.5 cm total length and 49.5 cm effective length was supplied by SGE (Melbourne, Australia). Detection wavelength was set at 214 nm. The sample injections were performed in a hydrodynamic mode (5 s under 0.5 psi).

### 2.2. Chemicals and Materials

All reagents were of analytical grade purity.

Borax, disodium hydrogen phosphate dihydrate, sodium dihydrogen phosphate monohydrate, potassium chloride, and CTAB (Cetyl trimethylammonium bromide) were from FLUKA (Buchs, Switzerland). Phosphoric acid, sodium hydroxide, chloric acid, and acetonitrile were supplied by Carlo Erba (Milan, Italy). Trizma base (Tris [hydroxymethyl]aminomethane), EDTA (ethylenediaminetetraacetic acid). Sodium bicarbonate, citric acid, oxalic acid, and sodium bitartrate monohydrate were from Sigma-Aldrich (Steinheim, Germany).

The real samples were commercially available pharmaceutical products: Berocca Plus (Bayer S.p.A), Gabbrovital B forte (CEVA VETEM SpA), Trinidex (LDB LAB.DIACO BIOMEDICALI SpA), and Biochetasi (Sigma-Tau SpA). For solution filtering the syringe filters 0.45 *μ*m (Millex HV, Millipore, MA, USA) were used.

#### 2.2.1. The Standards of Vitamins

thiamine hydrochloride (B_1_), thiamine pyrophosphate chloride (B_1a_), riboflavine (B_2_), riboflavine-5′monophosphate (B_2a_), nicotinamide (B_3_), d-pantothenic acid calcium salt (B_5_), pyridoxine hydrochloride (B_6_), biotin (B_8_), folic acid (B_9_), B_10_ (4-aminobenzoic acid), cyanocobalamin (B_12_), hydroxocobalamin (B_12a_), and 2-3 dihydroxybenzoic acid were purchased from Fluka (Buchs, Switzerland).

### 2.3. Standard, Buffer, and Real Sample Preparation

Standard stock solutions of studied analytes (at 1 mg/mL concentration) were prepared in distilled water, riboflavine and folic acid were dissolved in HCl, respectively, 3 M and 2 M.

The calibration solutions were obtained by dilution of the stock solution with distilled water to give a desired analyte concentration. The real samples preparation was different for each product.

For Berocca Plus preparation, twenty tablets were weighted and grounded in a mortar. The quantity of powder equivalent at one tablet was weighted and dissolved in a 50 mL volumetric flask with distilled water. After sonication for 10 min in an ultrasonic bath, the supernatant solution was centrifuged for 5 min and then filtered through the 0.45 *μ*m syringe filter. Finally the solution was diluted with distilled water to reach a concentration order inside the calibration range.

For Trinidex sample, the intravenous injection solution was filtered through the 0.45 *μ*m syringe filter without dilution and injected.

For Gabbrovital preparation, the veterinary intramuscular injection solution was diluted 1 : 1000 because of its high concentration, then filtered and analysed.

For Biochetasi sample, the powder contained in the vial for the intramuscular injection, was diluted with distilled water to reach a desired concentration and then filtered through the 0.45 *μ*m syringe filter.

## 3. Results and Discussion

### 3.1. Method Development and Optimization

In order to propose a suitable method for routine analysis, it was necessary to evaluate the experimental conditions for the best resolution of studied analytes.

First, because of different solubility and stability for each vitamin, many conditions for their dissolution were tested. Several parameters for the CZE analysis, such as BGE composition and concentration, pH, injection conditions, wavelengths, applied voltage, and temperature were explored.

#### 3.1.1. Effect of BGE Composition, Concentration, and pH

The BGE buffer composition is one of the most important parameters in capillary electrophoresis.

In order to obtain maximum precision level, it is necessary to choose the buffer very carefully.

Most of vitamins are nitrogen-containing, protonable molecules, so starting point of this work was to explore the low range of pH values for their separation.

In the preliminary step of study, the BGEs tested pH ranging between 1.5–4.2 and concentration ranging between 20 mM to 50 mM, are HCl/KCl, phosphate, phosphate-oxalate, phosphate with 5% acetonitrile, phosphate with CTAB, phosphate-tartrate, and tartrate.

**Figure 1:**
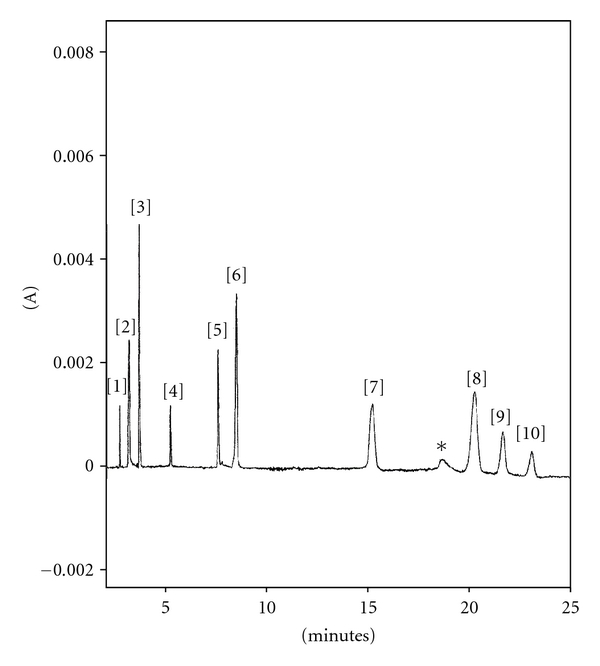
Electropherogram of standard mixture in acidic BGE. Peak identification: (1) B_1_ 20 *μ*g/mL; (2) B_3_ 10 *μ*g/mL; (3) B_6 _5 *μ*g/mL; (4)* B *_12a_ 20 *μ*g/mL; (5)* B *_1a_10 *μ*g/mL; (6) B_10_ 5 *μ*g/mL; (7) B_9_ 15 *μ*g/mL; (8) B_2_ + B_12_ (10 + 20) *μ*g/mL; (9) B_7_ 10 *μ*g/mL; (10) B_5_ 40 *μ*g/mL. Experimental conditions: phosphate buffer 20 mM; pH = 2.3;  *V* = 30 kV; *i* = 80 *μ*A; *T* = 25°C; injection at 0.5 psi for 5 s, fused silica capillary i.d. = 50 *μ*m, *L* = 59.5 cm, *l* = 49.5 cm instrument P/ACE MDQ System (Beckman).

Only in 20 mM phosphate buffer at pH = 2.3 good separation of eleven analytes was realised in about 23 minutes. In Figure 1, analytes from 1 to 7 are cations, the others (8–10) are in anionic form. In these operating conditions, riboflavine (B_2_) and cyanocobalamin (B_12_) are overlaid. Since these compounds are often contained in the same pharmaceutical preparation, it is impossible to determinate both of them. Long analysis time, high intensity of current, and low repeatability of migration time (RSD% ~ 10%) did not allow to propose this procedure for routinely analyses.

Consequently the possibility of separation of studied vitamins in alkaline buffers were evaluated.

BGE buffers at pH between 8.5 and 9.3 into the range of concentration 20 mM to 80 mM were explored: tris/HCl, bicarbonate/carbonate, tris/borate, borate, borate with EDTA, and borate with CTAB.

**Figure 2:**
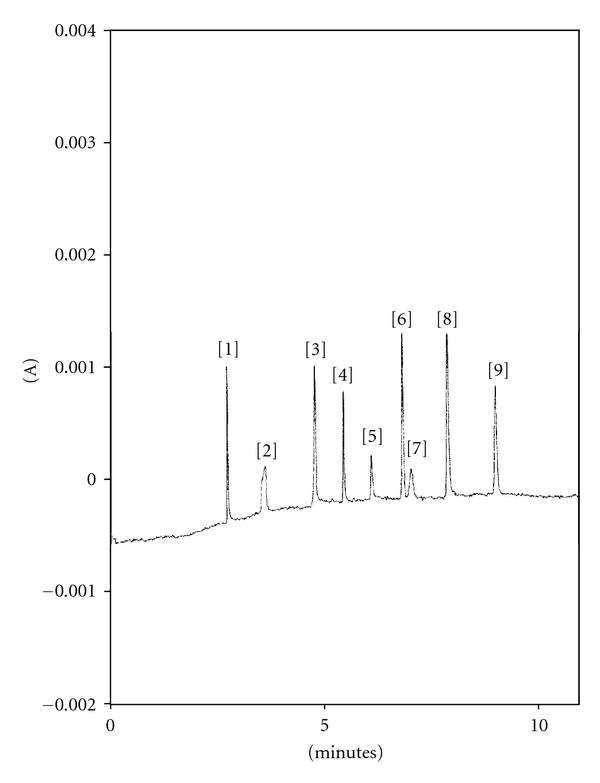
Electropherogram of standard mixture in basic BGE. Peak identification: (1) B_1_ 20 *μ*g/mL; (2) B_3_ 10 *μ*g/mL; (3) B_2_ 10 *μ*g/mL; (4) B_6_ 5 *μ*g/mL; (5) B_1a_ 10 *μ*g/mL; (6) B_10_ 5 *μ*g/mL; (7) B_5_ + B_2a_ (40 + 10) *μ*g/mL; (8) S.I. 3 *μ*g/mL; (9) B_9_ 20 *μ*g/mL. Experimental conditions: borate buffer 20 mM; pH = 9.2; *V* = 20 kV; *i* = 25 *μ*A; *T* = 25°C; *t*_*inj*_ = 5 s *p*_*inj*_ = 0.5 psi fused silica capillary ID = 50 *μ*m; *L* = 59.5 cm; *l* = 49.5 cm, instrument P/ACE MDQ System (Beckman).

Best separation of nine vitamins was realized in 20 mM borate buffer at pH 9.2 in about 9 minutes (Figure 2).

The peaks are symmetric and baseline resolved for individual B vitamins with exception of d-pantothenic acid calcium salt (B_5_) and riboflavine-5′monophosphate (B_2a_), that are coeluted. Since a good compromise between peak symmetry, resolution, and analysis time resulted in 20 mM borate buffer at pH = 9.2, these experimental conditions were chosen as optimal.

#### 3.1.2. Effect of Wavelength

Primarily it was necessary to select the most suitable wavelength for the simultaneous detection of vitamins, since spectral properties of each one differs enough. The UV spectra obtained by UV-DAD show three suitable wavelengths for quantitative analysis. 214 nm for all the vitamins, at exception of d-pantothenic acid calcium salt (B_5_) and riboflavine-5′monophosphate (B_2a_), that are overlaid. Then the quantitative analysis of these two analytes was realized at the wavelengths of 190 and 260 nm, because the considered compounds do not interfere themselves. In fact vitamin B_5_ has only maximum of absorbance at 190 nm, while vitamin B_2a_ presents two maximums at 214 and 260 nm.

#### 3.1.3. Effect of Applied Voltage

For the optimization of separation voltage the analyses ranging the applied voltage between 10 and 30 kV were carried out. At low voltage the separation of all the analytes was reached but the analysis time increased to 20 min with the broadening of peaks. Increasing the voltage (max 30 kV) the optimal value results in 20 kV producing the current intensity of 25 uA and good repeatability. At 30 kV the separation of analytes was incomplete.

#### 3.1.4. Effect of Temperature

The increase of temperature (30°C) produced the shortening of analysis time (the viscosity of the BGE decrease). It should be advantageous setting high temperature of cartridge but B-complex is thermolabile. So the suitable temperature for analyses of these compounds resulted in 25°C.

### 3.2. Method Validation

The method was validated following the ICH guideline [20].

#### 3.2.1. Precision

The precision was evaluated in terms of RSD% of migration time (*t*_*m*_) and corrected peak area (*A*_*c*_) for intraday and interday analyses. For the intra-day precision evaluation the standard solutions at three concentrations levels (low, intermediate, and high) were injected in the same day. To estimate the interday precision, the standard solutions were analysed for five consecutive days performing five consecutive injections every day. The calculated RSD% values are reported in Table 1. 

#### 3.2.2. Calibration Range

The linearity of detector response was tested in different ranges as reported in Table 2. For the quantitative analysis the Internal Standard method was applied. For its property, was choosen Internal Standard 3,4 dihydroxy-benzoic acid.

Six standard solutions, containing nine analytes of interest and the Internal Standard, were injected in triplicate. The calibration lines were obtained plotting R (corrected areas ratio) versus the standard solutions concentration using Microsoft EXCEL.

#### 3.2.3. LOD and LOQ

LOD, the lowest concentration of analytes that can be distinguished from the noise, defined as signal to noise ratio S/N of 3 : 1 was ranging between 0.9 *μ*g/mL to 9.0 *μ*g/mL.

LOQ, the lowest concentration of analytes that can be quantified with good precision, defined as signal to noise ratio S/N of 10 : 1 was ranging between 3.0 *μ*g/mL to 30.0 *μ*g/mL.

#### 3.2.4. Analysis of Real Samples

**Figure 3:**
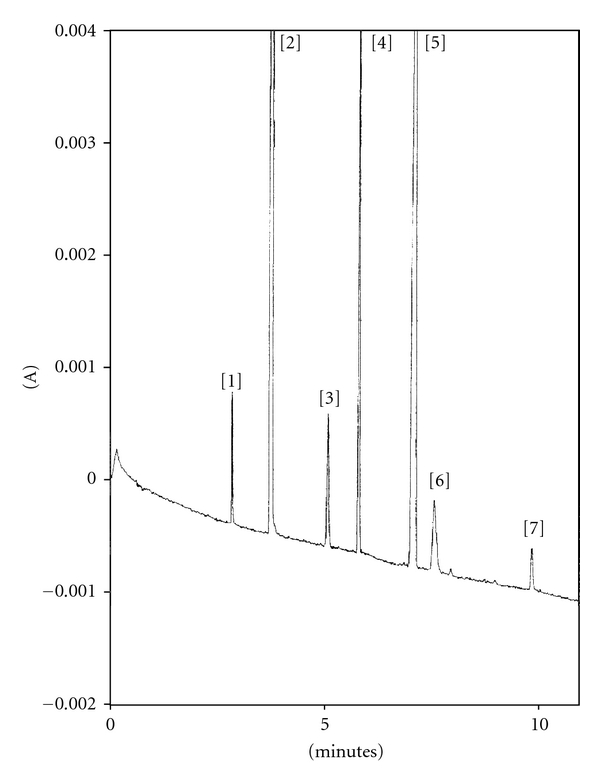
Electropherogram of real sample Berocca Plus. Experimental conditions: see Figure 2. Peak identification: (1) B_1_ 31 *μ*g/mL; (2) B_3_ 36 *μ*g/mL; (3) B_2_ 35 *μ*g/mL; (4) B_6_ 40 *μ*g/mL; (5) ascorbic acid (not determined); (6) B_9_ 5 *μ*g/mL; (7) SI 3 *μ*g/mL. The concentrations are the nominal values.

Four pharmaceutical formulation containing B-complex vitamins were analyzed. The experimental results are given in Table 3. No interference was observed in pharmaceutical solutions. RSD% of migration time and corrected peak area values were lower, respectively, than 0.5% and 3.0%. A typical electropherogram of commercial pharmaceutical preparation (Berocca Plus) is shown in Figure 3.

#### 3.2.5. Accuracy

Accuracy was evaluated by recovery assays on the commercial product. After fortification, the real sample undergo the analysis and the resulted concentration were compared with the data obtained without fortification. The resulting recoveries data are ranging from 97.0 to 101.4%.

#### 3.2.6. Ruggedness

**Figure 4:**
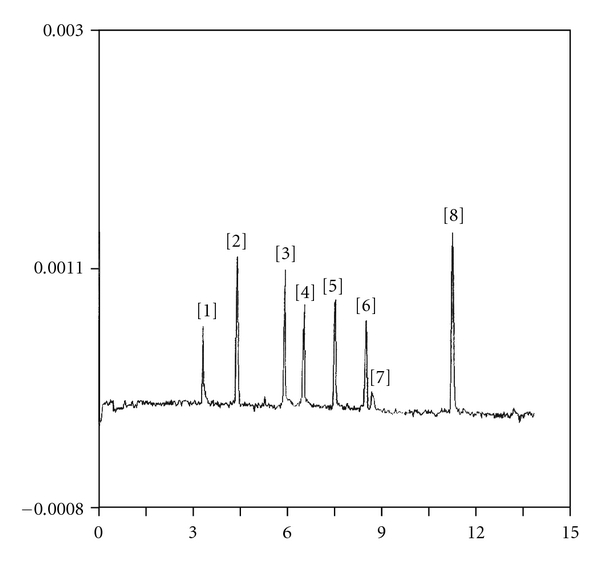
Electropherogram of standard mixture. Peak identification: (1) B_1_, (2) B_3_, (3) B_2_, (4) B_6_, (5) B_1a_, (6) B_10_, (7) B_5_ + B_2a_, (8) B_9._ The vitamin concentrations are the same as in Figure 2. Experimental conditions: borate buffer 20 mM; pH = 9,2; *V* = 10 kV; *i* = 17 *μ*A; *T* = 25°C; hydrodynamic injection *t*_*inj*_ = 1 s; fused silica capillary ID = 50 *μ*m; *L* = 44 cm; *l* = 36 cm; instrument Spectra Phoresis 1000.

For this purpose, measurements on the SpectraPhoresis 1000 apparatus in the optimal experimental conditions were performed. In this instrument the cartridge form is different and the capillary total length is 44 cm, the effective length 36 cm (shorter than optimal length of capillary utilized on the Beckman instrument). Consequently, on this instrument, it was necessary to apply lower voltage (10 kV). The electropherogram, obtained on this apparatus, injecting the standard mixture in hydrodynamic mode for 1 sec, is shown in Figure 4. Repeatability of migration time is ranging from 0.32% to 0.52%.

## 4. Conclusion

Proposed method was developed for the simultaneous determination of nine water-soluble vitamins (B_1_, B_1a_, B_2_, B_2a_, B_3_, B_5_, B_6_, B_9_, and B_10_) in the multivitamin pharmaceutical formulations by CZE. The best resolution was obtained in about 9 min using a simple tetraborate buffer 20 mM, pH = 9.2 at 25°C, and constant voltage of 20 kV in uncoated fused silica capillary. The method is suitable for the vitamins B analysis as the validation confirmed: it is precise, accurate, and rugged. The analyses of real samples proved its applicability. Since no expensive reagent and no pretreatment of samples are required in this procedure, it offers a valid alternative for quality control analysis in the pharmaceutical industry. 

## Figures and Tables

**Table 1:** Intraday and interday precision of migration time (*t*_*m*_) and corrected peak area (*A*_*c*_) in optimal condition at low-, intermediate-, and high-concentration levels.

	RSD%		RSD%			
	*t*_*m*_		*A*_*c*_			
	Intraday	Interday	Intraday		Interday	
			Low	Intermediate	High	Intermediate
B_1_	0.20	0.31	5.51	2.89	1.76	4.37
B_3_	0.17	0.57	3.16	2.54	2.30	4.31
B_2_	0.24	0.99	4.89	1.89	1.41	4.15
B_6_	0.32	0.77	5.48	2.39	1.91	3.22
B_1a_	0.34	0.81	6.29	1.95	1.26	3.87
B_10_	0.32	0.95	3.83	1.91	2.14	4.28
B_5_	0.27	0.87	4.91	1.04	1.56	3.17
B_2a_	0.34	0.57	5.00	2.59	1.51	4.91
SI	0.34	0.82	4.51	2.15	1.99	2.61
B_9_	0.38	1.01	5.74	1.16	2.84	4.33

**Table 2:** Method linearity data.

Analyte	Calibration range *μ*g/mL	Equation	*r* ^2^
B_1_	5–200	*y* = 0.0235*x* − 0.0111	0.9952
B_3_	10–120	*y* = 0.0722*x* − 0.2297	0.9931
B_2_	5–40	*y* = 0.0742*x* − 0.0104	0.9989
B_6_	3–40	*y* = 0.0597*x* − 0.0024	0.9993
B_1a_	5–40	*y* = 0.0183*x* − 0.0194	0.9956
B_10_	3–25	*y* = 0.0887*x* − 0.0240	0.9973
B_5_	30–120	*y* = 0.0077*x* − 0.0666	0.9970
B_2a_	5–40	*y* = 0.0224*x* + 0.0053	0.9972
B_9_	5–30	*y* = 0.0557*x* − 0.0488	0.9912

**Table 3:** Commercial product analysis.

Commercial product	Berocca plus		Gabbrovital B		Trinidex		Biochetasi	
Analyte	mg/tab		mg/100 mL		mg/100 mL		mg/vial	
	Found	Declared	Found	Declared	Found	Declared	Found	Declared

B_1_	1.55 ± 0.06	1.54	15.24 ± 0.38	15.00	0.98 ± 0.05	1.00	—	—
B_1a_	—	—	—	—	—	—	40.41 ± 1.20	47.00
B_2_	1.77 ± 0.36	1.76	—	—	—	—	—	—
B_2a_	—	—	—	—	—	—	31.10 ± 1.19	28.30
B_3_	17.93 ± 0.97	18.00	3.03 ± 0.17	3.00	11.03 ± 0.91	10.00	—	—
B_5_	6.48 ± 0.50	6.60	—	—	—	—	—	—
B_6_	2.18 ± 0.11	2.20	1.50 ± 0.03	1.50	20.18 ± 0.24	20.00	12.35 ± 0.23	15.00
B_9_	0.24 ± 0.03	0.25	—	—	—	—	—	—
